# Supplementary material for: Dissecting the eQTL Micro-Architecture in Caenorhabditis elegans
Source: Front Genet. 2020 Nov 3;11:501376. doi: 10.3389/fgene.2020.501376 (PMC7670075; doi:10.3389/fgene.2020.501376)
Supplement: Supplementary Figure 1 — Coverage per locus and per QTL. (A) The coverage in CB4856 loci per location on the genome, split out for ILs and RILs. The 56 ILs together have a higher coverage over the chromosome arms, where also most QTL map. The 48 RILs have a more homogenous distribution, only at the peel-1/zeel-1 locus on chromosome I there is low coverage (Seidel et al., 2008). (B). A histogram of the number of CB4856 loci covering an eQTL. Typically, an eQTL is covered by CB4856 loci of 2 ILs and 23 RILs (median). [file Presentation_1.zip › Supplementary Figure 5.PPTX]

## Slide 1
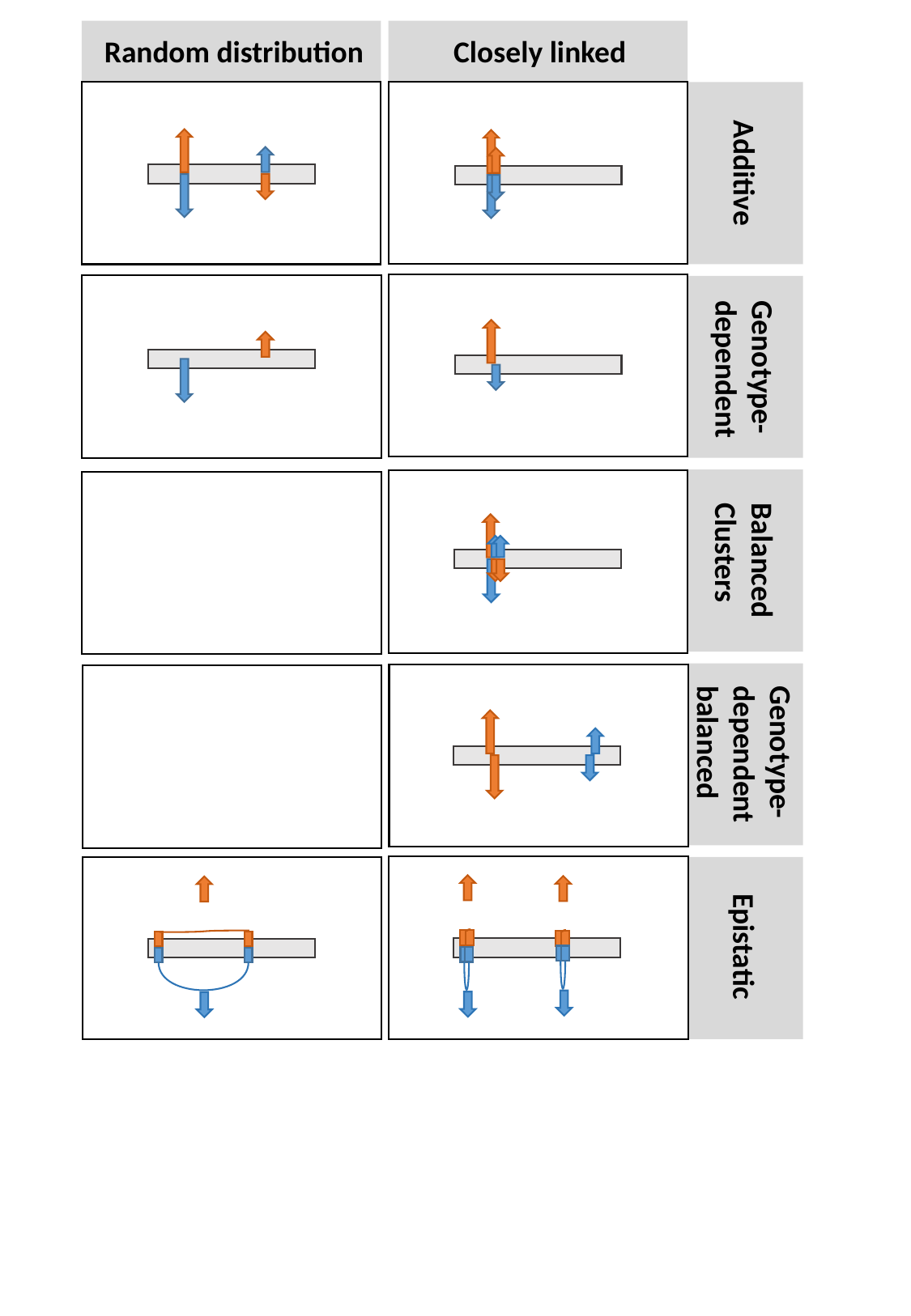

Closely linked
Random distribution
Additive
Genotype-
dependent
Balanced
Clusters
Genotype-
dependent
balanced
Epistatic
