# Supplementary figures and images for: Dissecting the eQTL Micro-Architecture in Caenorhabditis elegans
Source: Front Genet. 2020 Nov 3;11:501376. doi: 10.3389/fgene.2020.501376 (PMC7670075; doi:10.3389/fgene.2020.501376)

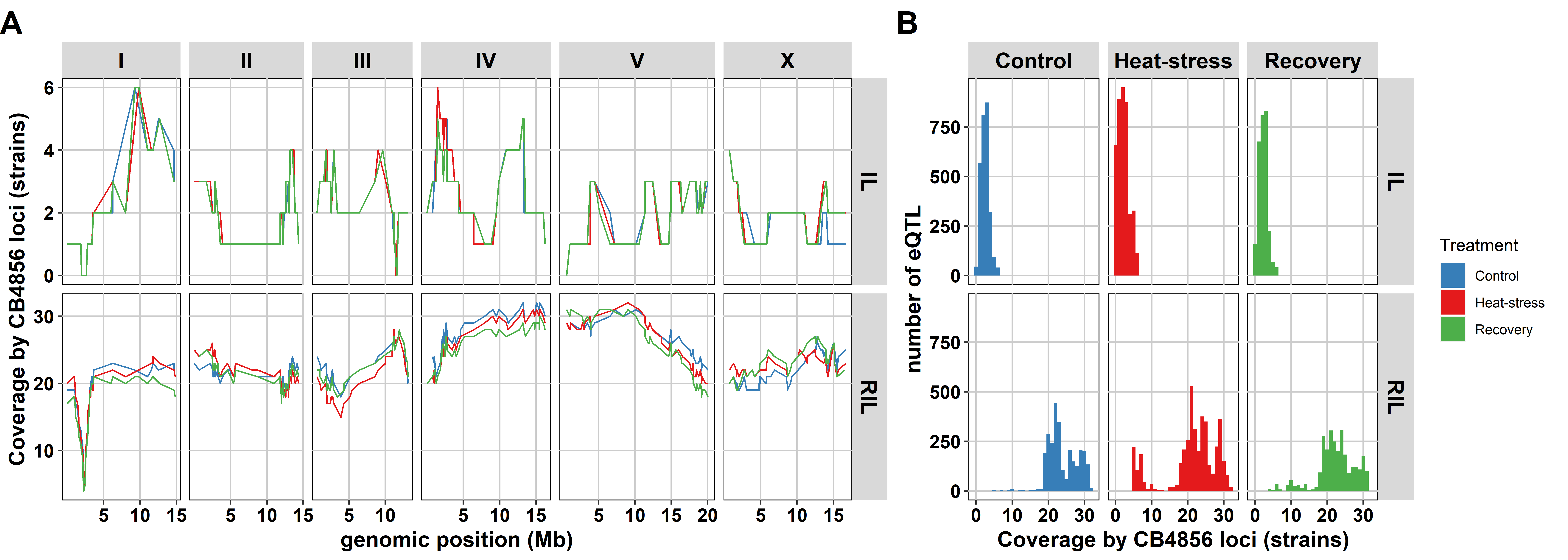

Supplement: Supplementary Figure 1 — Coverage per locus and per QTL. (A) The coverage in CB4856 loci per location on the genome, split out for ILs and RILs. The 56 ILs together have a higher coverage over the chromosome arms, where also most QTL map. The 48 RILs have a more homogenous distribution, only at the peel-1/zeel-1 locus on chromosome I there is low coverage (Seidel et al., 2008). (B). A histogram of the number of CB4856 loci covering an eQTL. Typically, an eQTL is covered by CB4856 loci of 2 ILs and 23 RILs (median). [file Presentation_1.zip › Supplementary Figure 1.TIF]

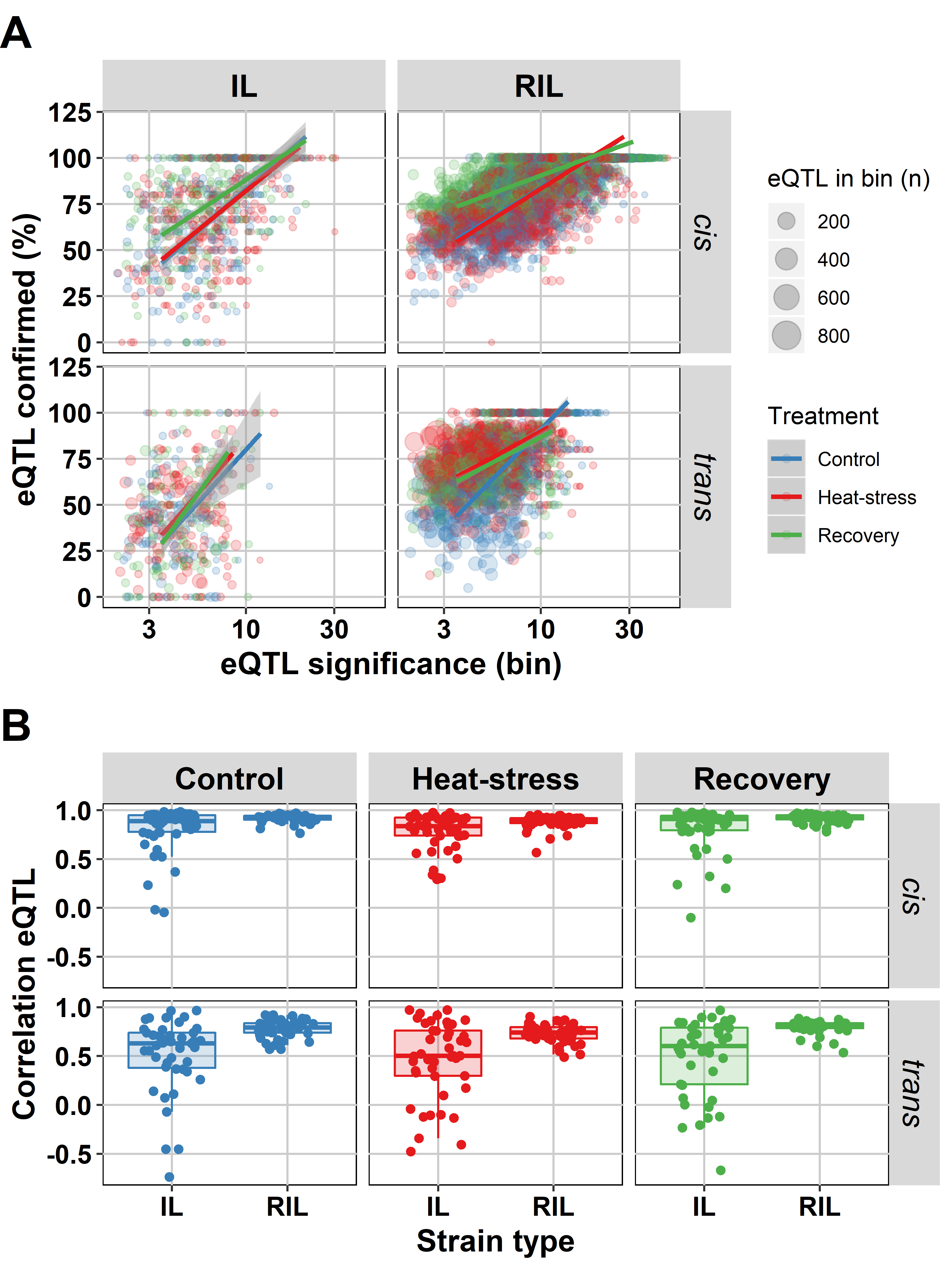

Supplement: Supplementary Figure 1 — Coverage per locus and per QTL. (A) The coverage in CB4856 loci per location on the genome, split out for ILs and RILs. The 56 ILs together have a higher coverage over the chromosome arms, where also most QTL map. The 48 RILs have a more homogenous distribution, only at the peel-1/zeel-1 locus on chromosome I there is low coverage (Seidel et al., 2008). (B). A histogram of the number of CB4856 loci covering an eQTL. Typically, an eQTL is covered by CB4856 loci of 2 ILs and 23 RILs (median). [file Presentation_1.zip › Supplementary Figure 2.TIF]

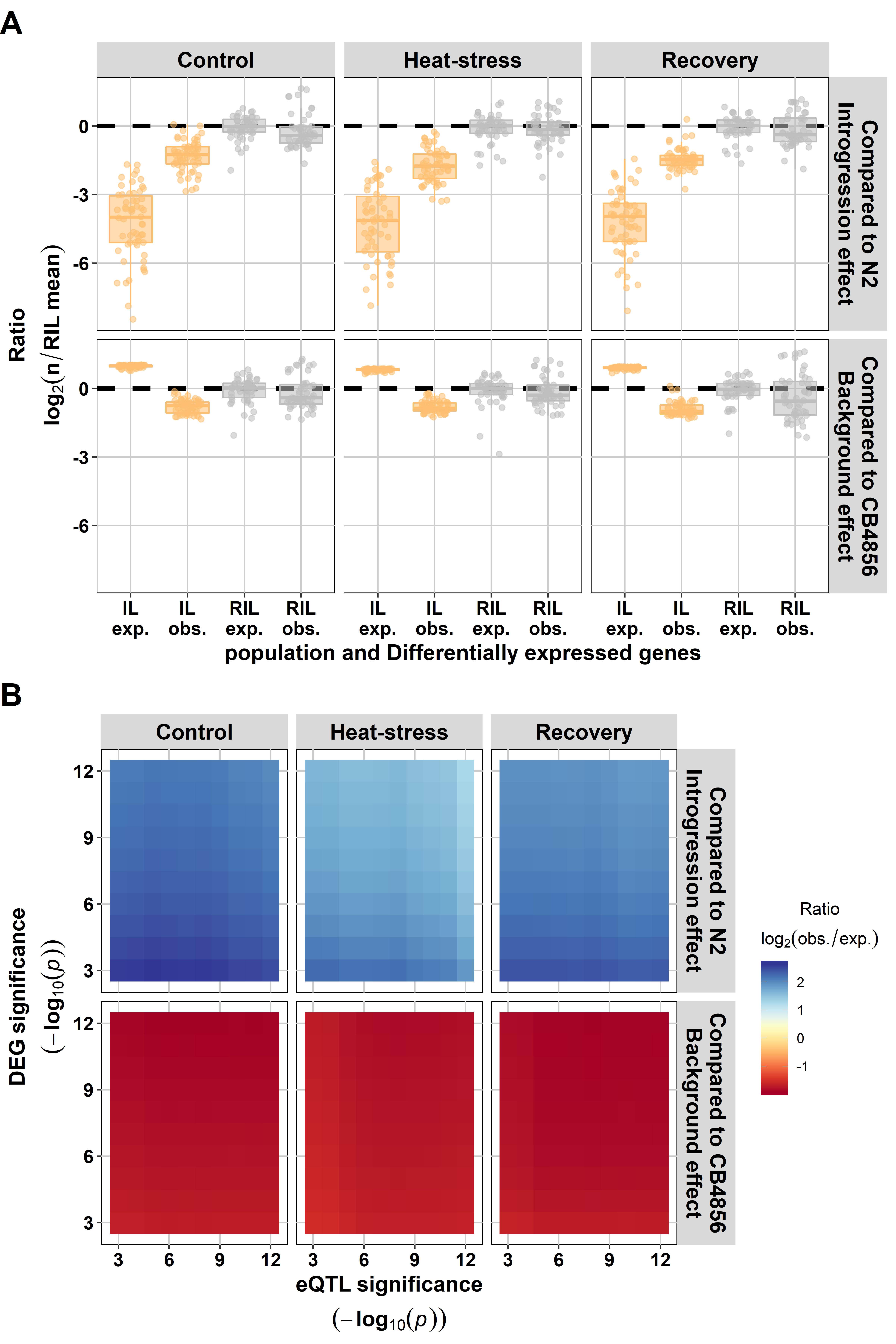

Supplement: Supplementary Figure 1 — Coverage per locus and per QTL. (A) The coverage in CB4856 loci per location on the genome, split out for ILs and RILs. The 56 ILs together have a higher coverage over the chromosome arms, where also most QTL map. The 48 RILs have a more homogenous distribution, only at the peel-1/zeel-1 locus on chromosome I there is low coverage (Seidel et al., 2008). (B). A histogram of the number of CB4856 loci covering an eQTL. Typically, an eQTL is covered by CB4856 loci of 2 ILs and 23 RILs (median). [file Presentation_1.zip › Supplementary Figure 3.TIF]

## Slide 1
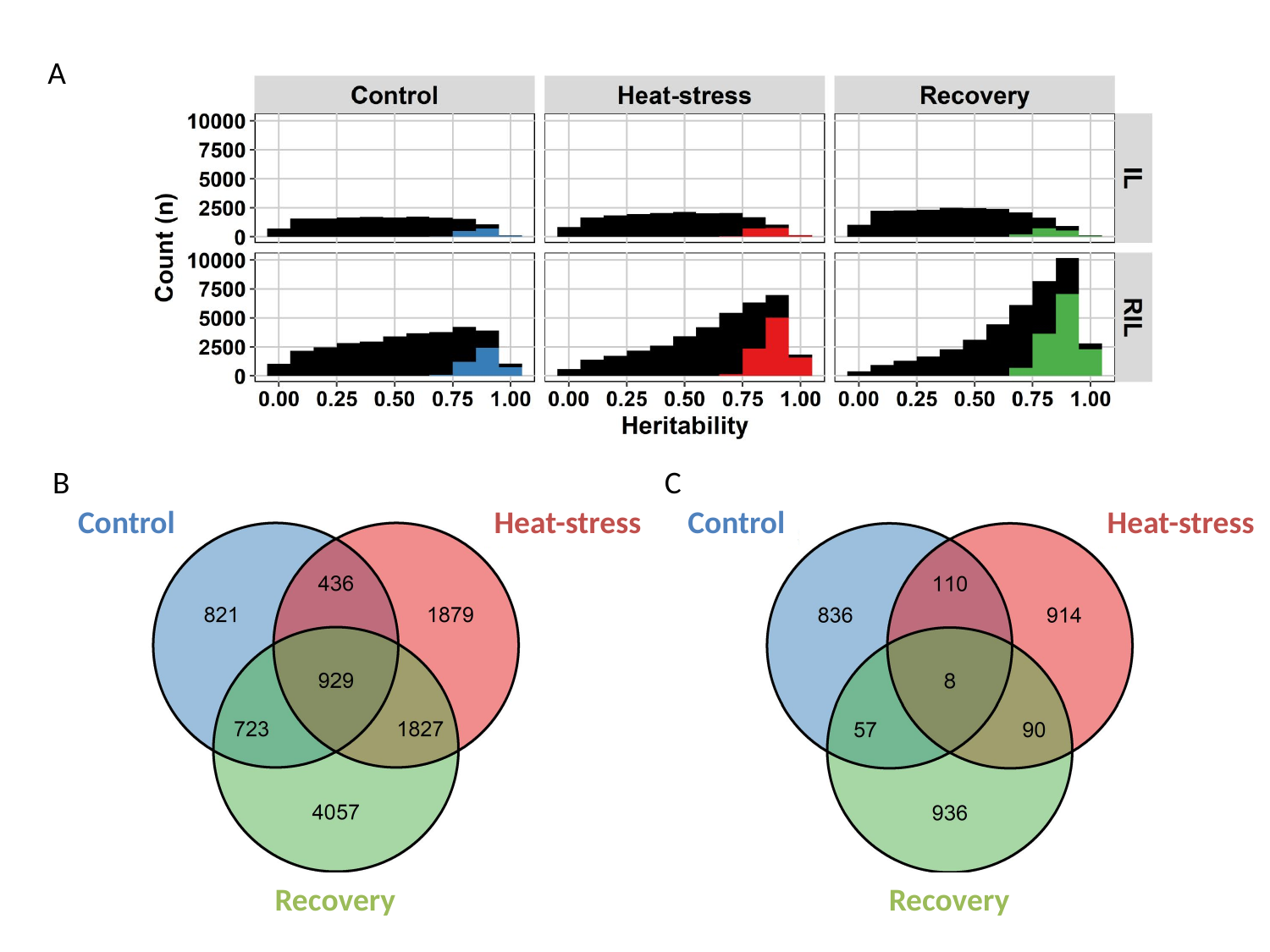

A
B
C
Control
Heat-stress
Control
Heat-stress
Recovery
Recovery

Supplement: Supplementary Figure 1 — Coverage per locus and per QTL. (A) The coverage in CB4856 loci per location on the genome, split out for ILs and RILs. The 56 ILs together have a higher coverage over the chromosome arms, where also most QTL map. The 48 RILs have a more homogenous distribution, only at the peel-1/zeel-1 locus on chromosome I there is low coverage (Seidel et al., 2008). (B). A histogram of the number of CB4856 loci covering an eQTL. Typically, an eQTL is covered by CB4856 loci of 2 ILs and 23 RILs (median). [file Presentation_1.zip › Supplementary Figure 4.PPTX]

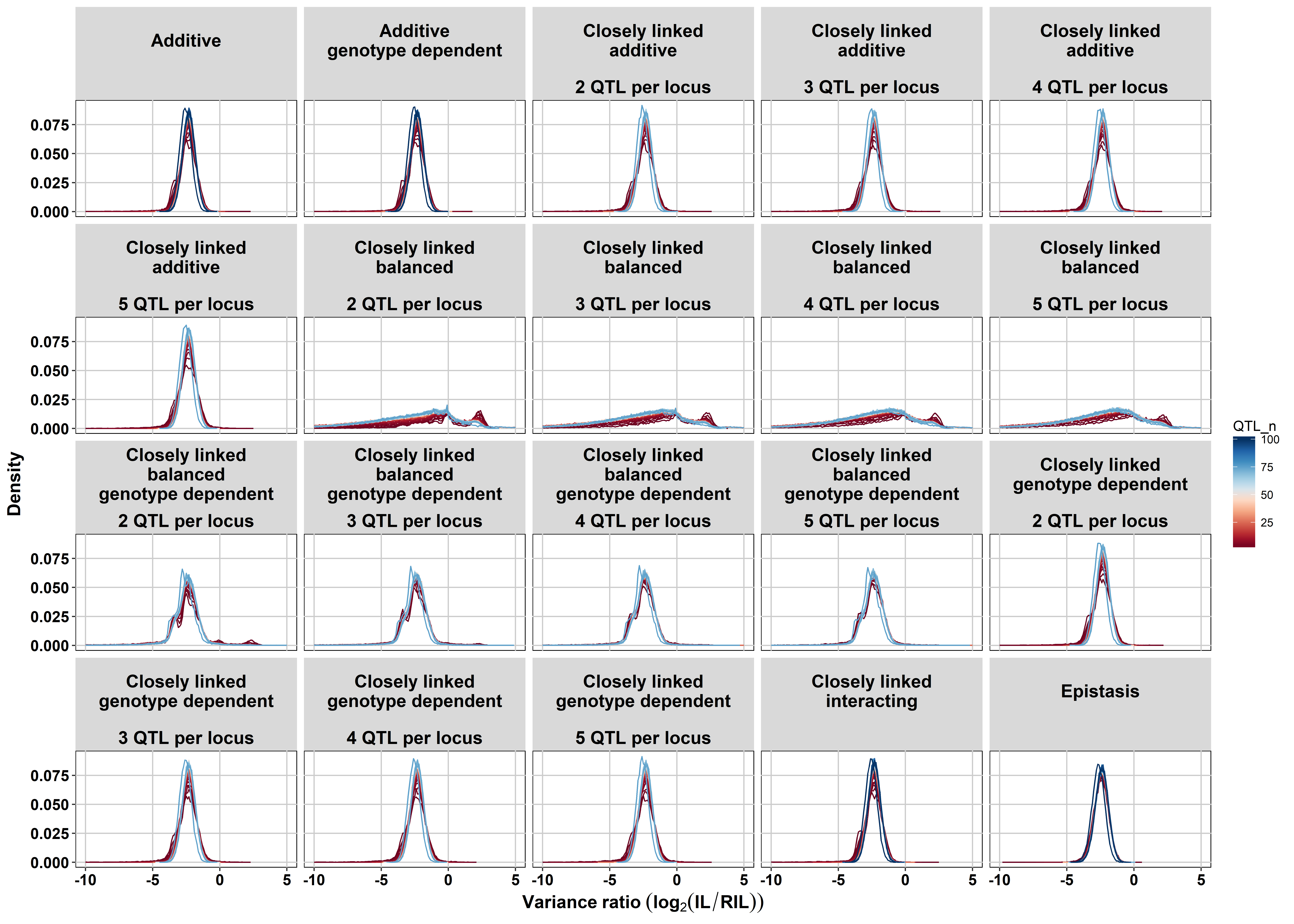

Supplement: Supplementary Figure 1 — Coverage per locus and per QTL. (A) The coverage in CB4856 loci per location on the genome, split out for ILs and RILs. The 56 ILs together have a higher coverage over the chromosome arms, where also most QTL map. The 48 RILs have a more homogenous distribution, only at the peel-1/zeel-1 locus on chromosome I there is low coverage (Seidel et al., 2008). (B). A histogram of the number of CB4856 loci covering an eQTL. Typically, an eQTL is covered by CB4856 loci of 2 ILs and 23 RILs (median). [file Presentation_1.zip › Supplementary Figure 6.TIF]

**A**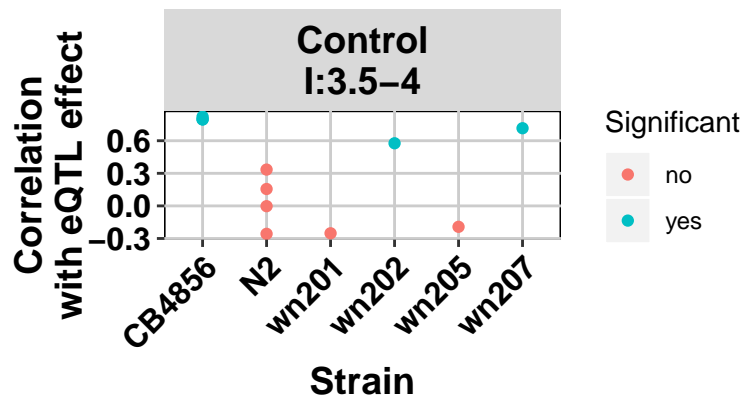**B**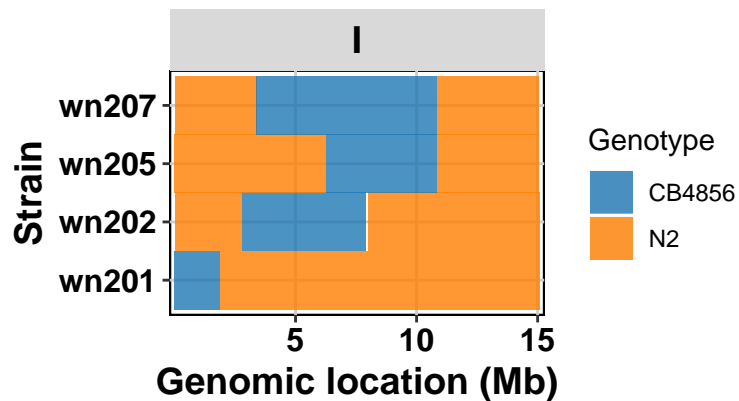**C**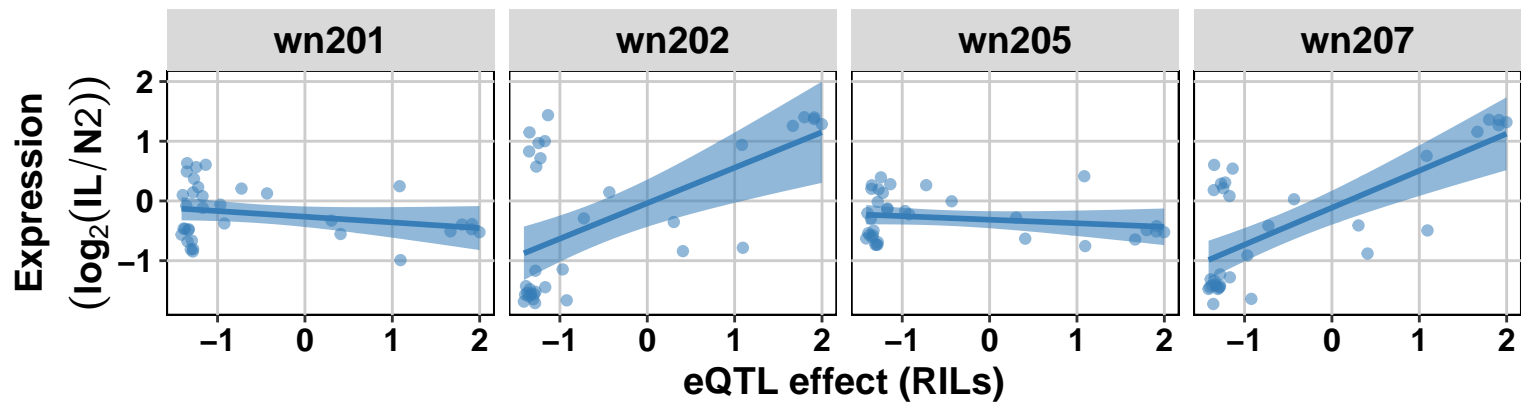

**A**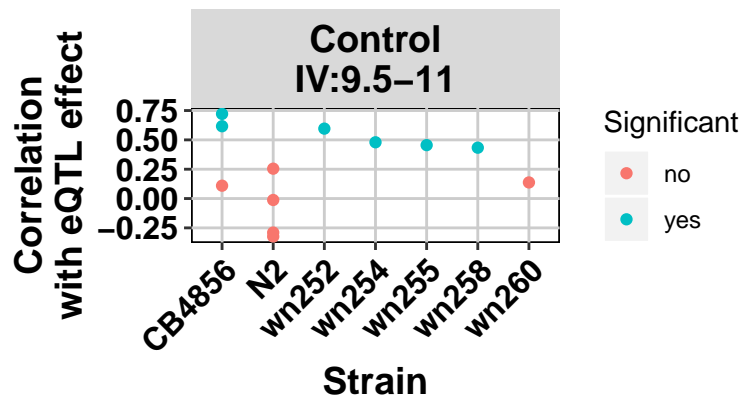**B**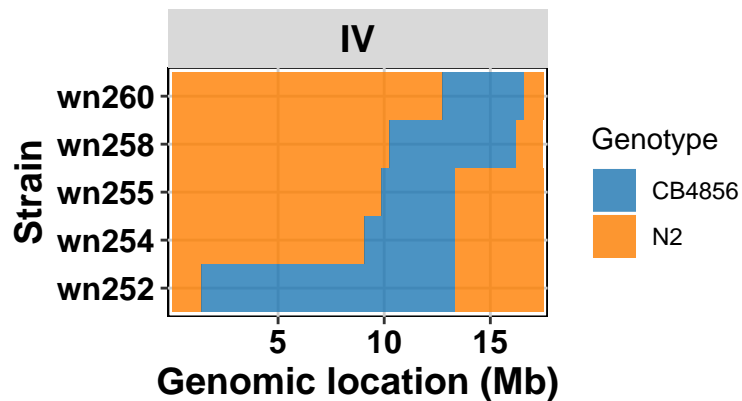**C**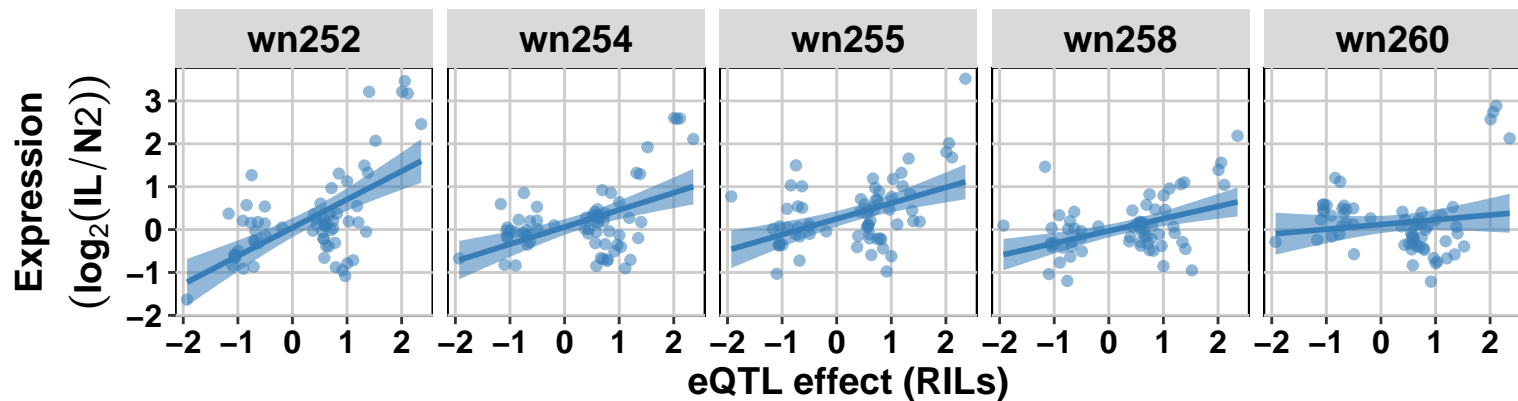

**A**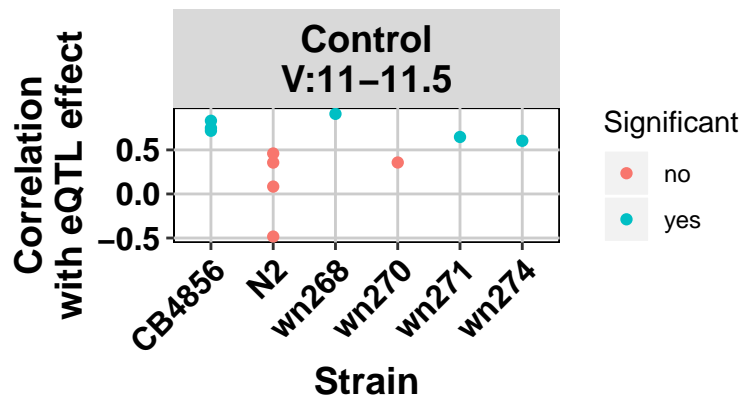**B**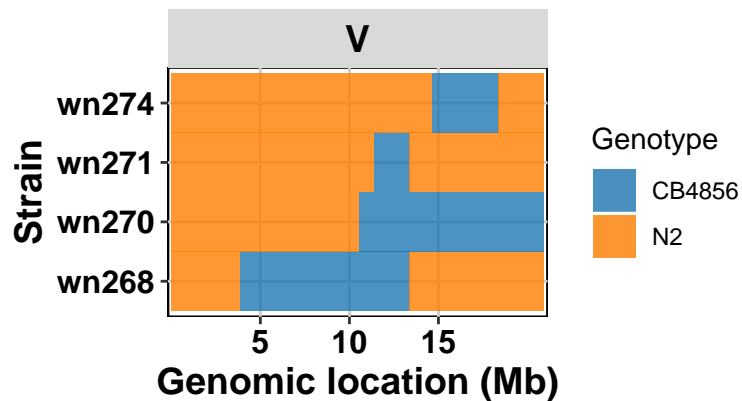**C**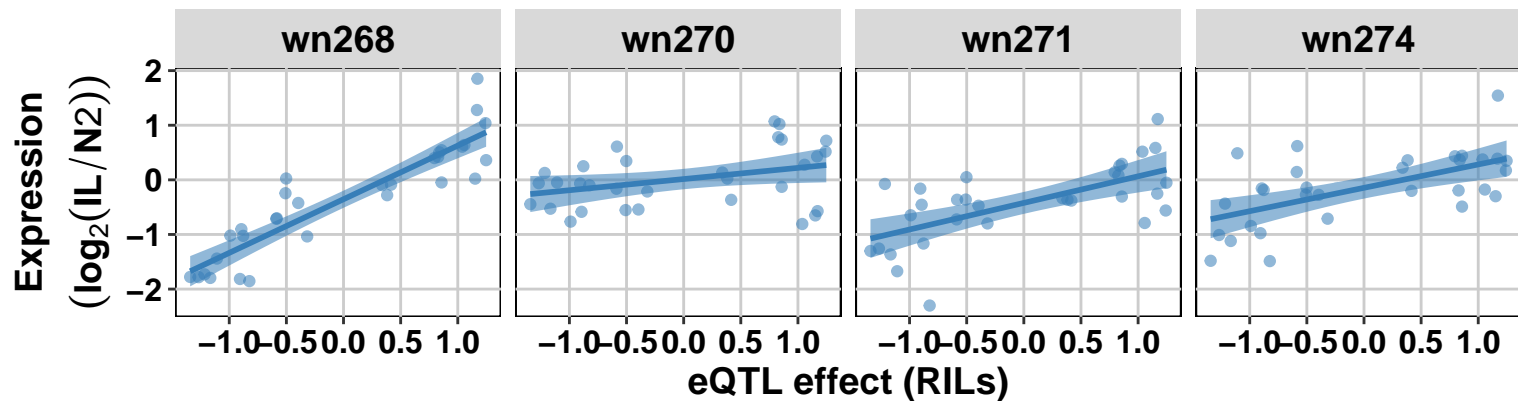

**A**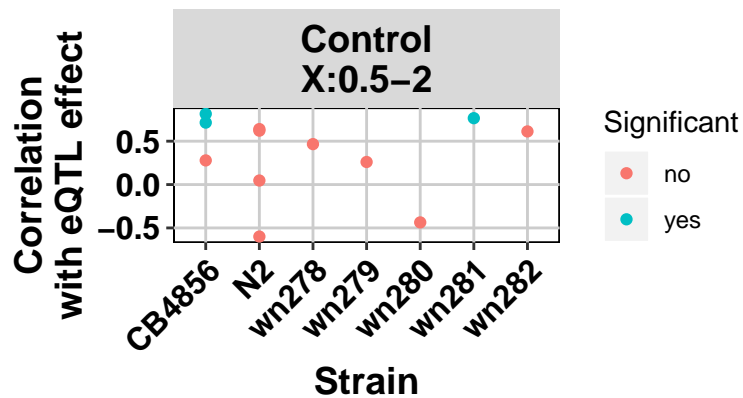**B**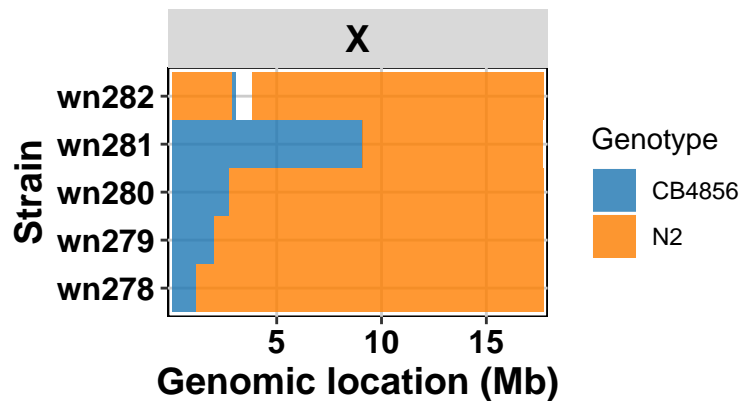**C**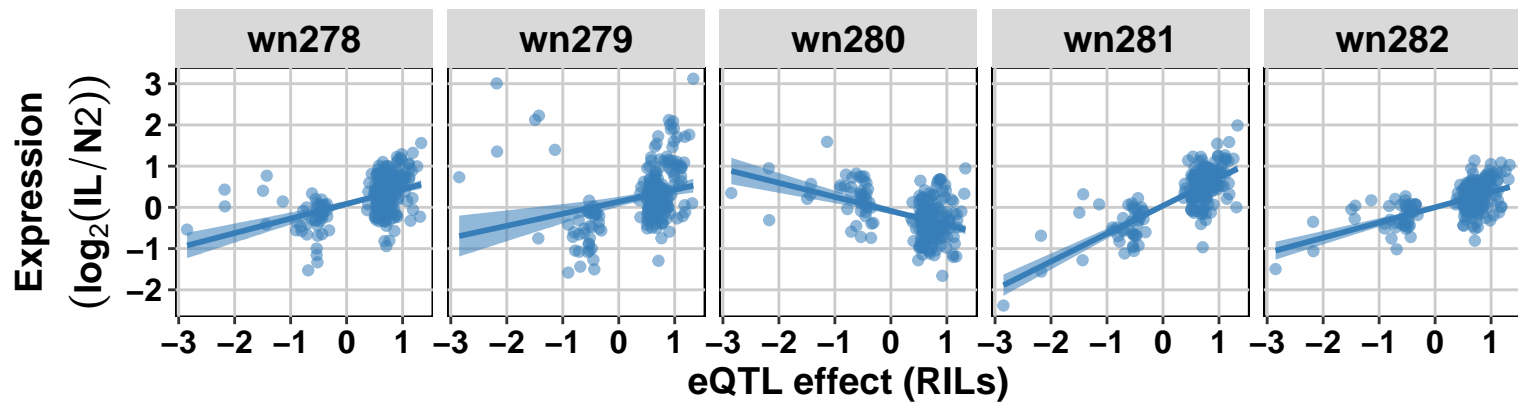

**A**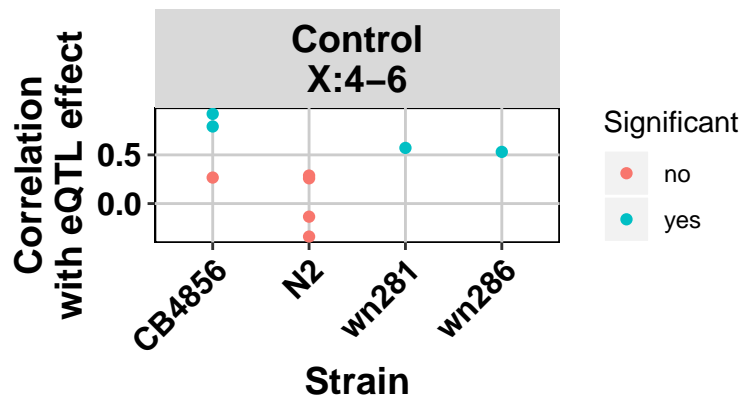**B**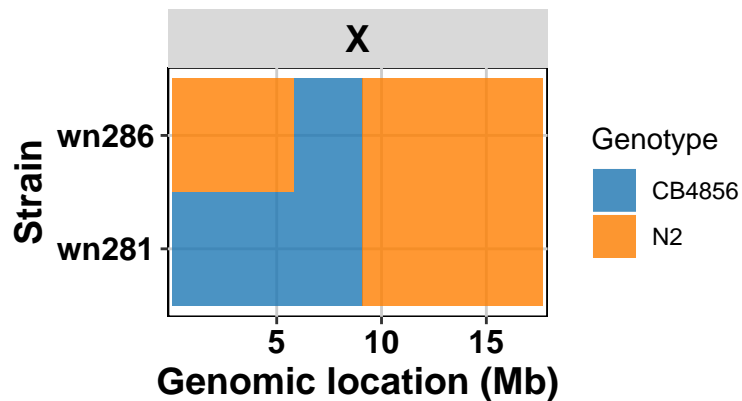**C**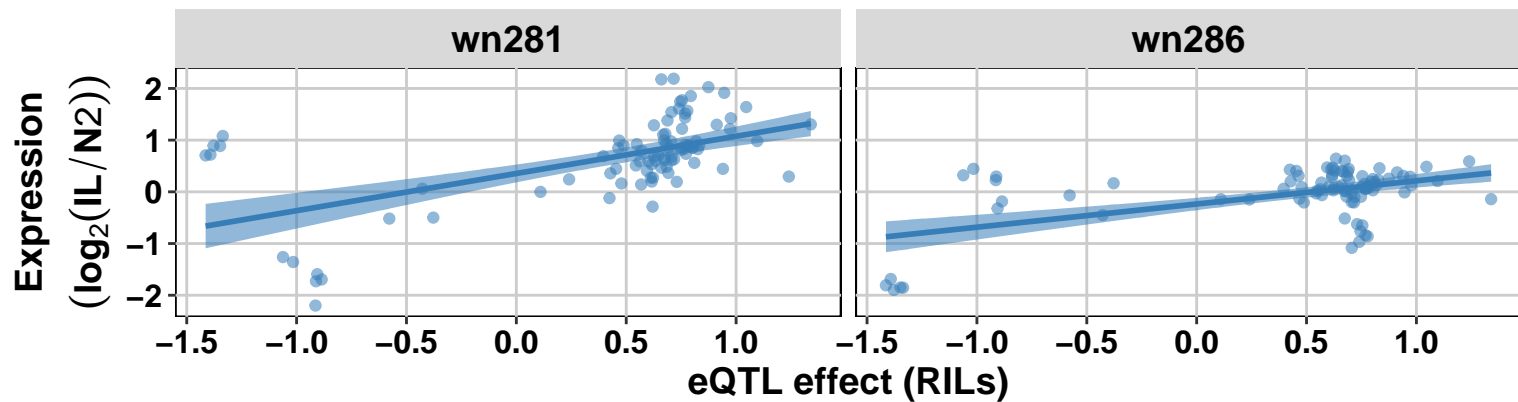

**A**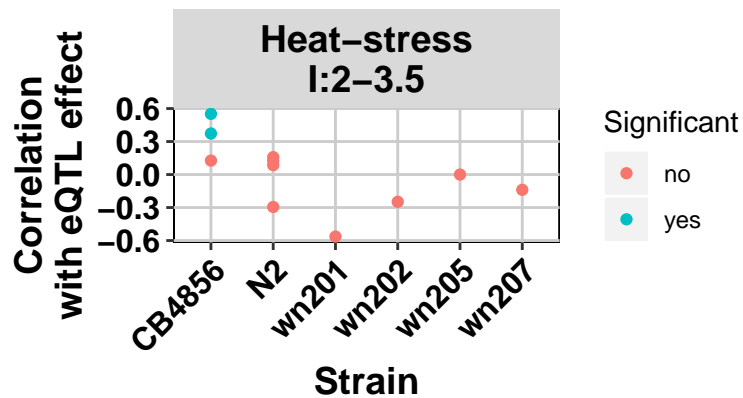**B**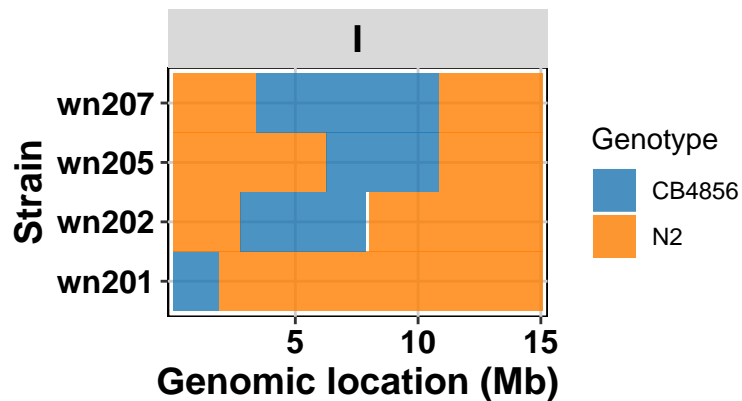**C**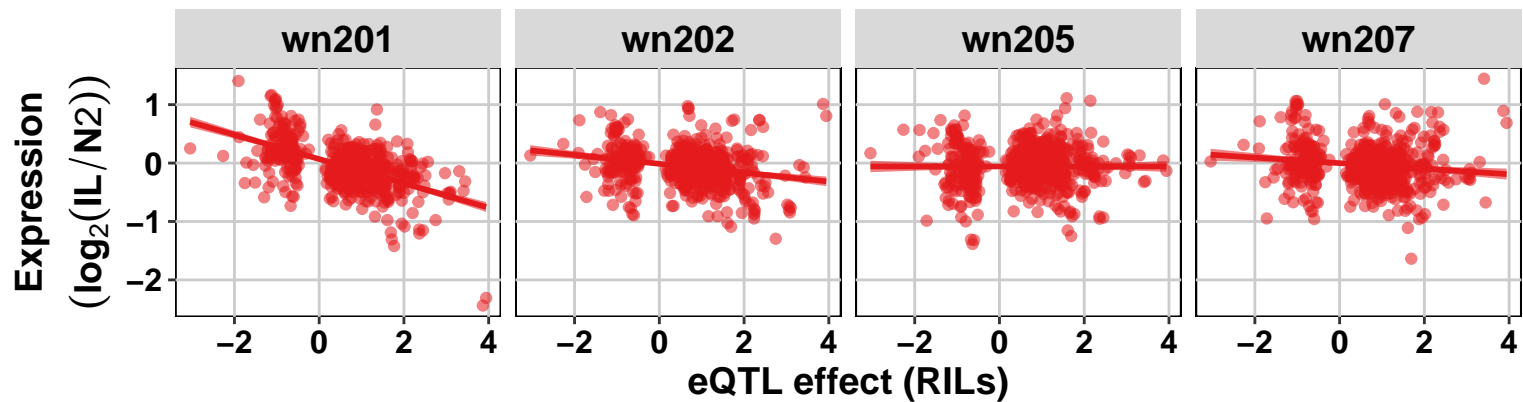

**A**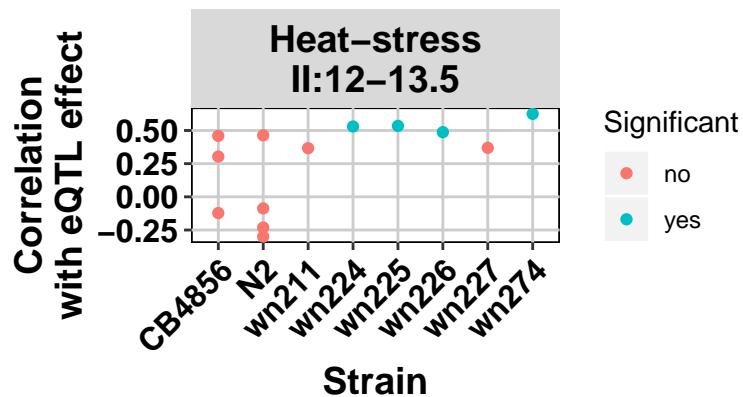**B**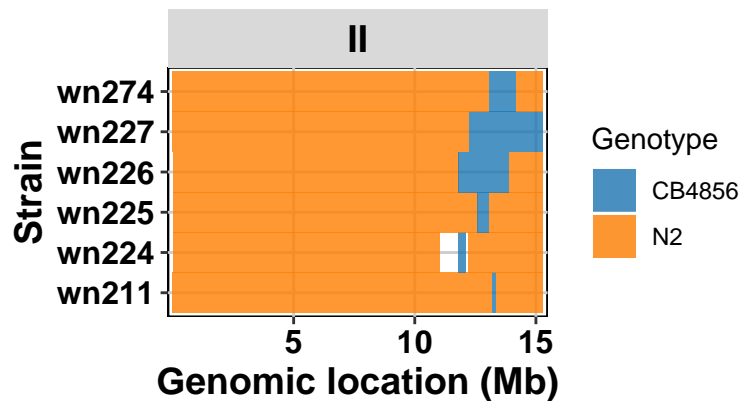**C**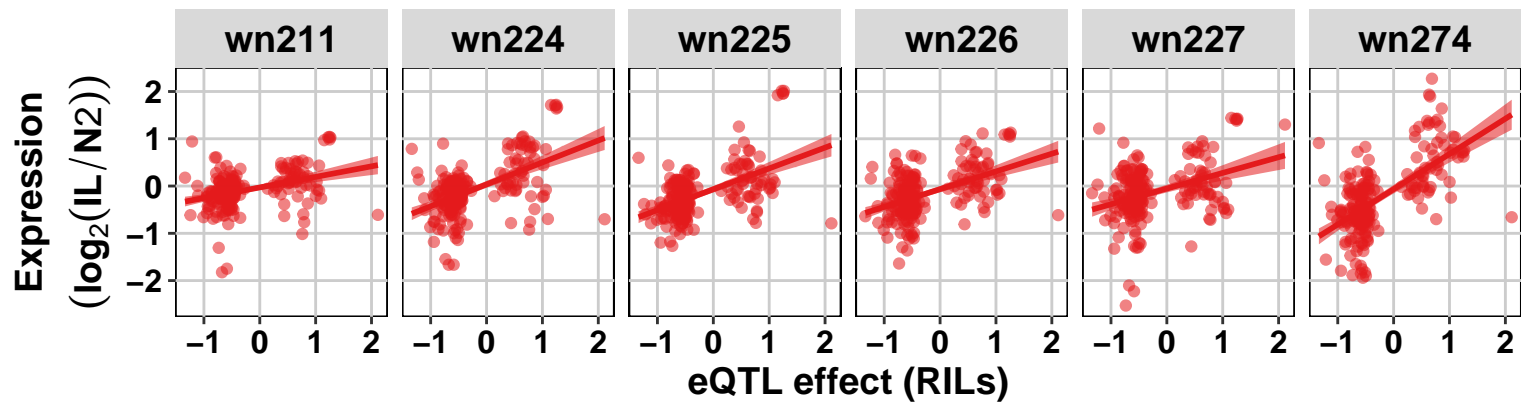

**A**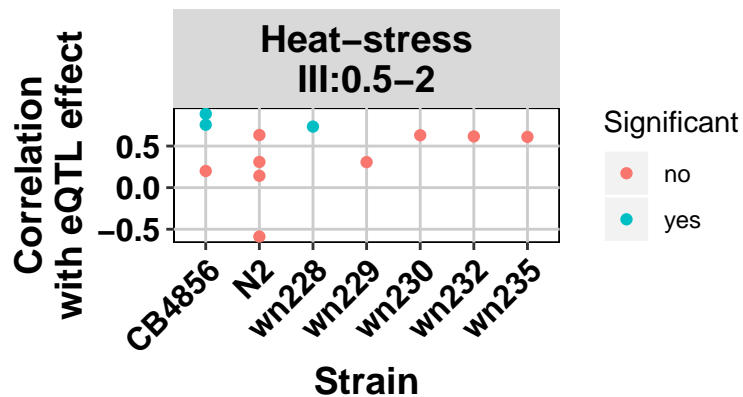**B**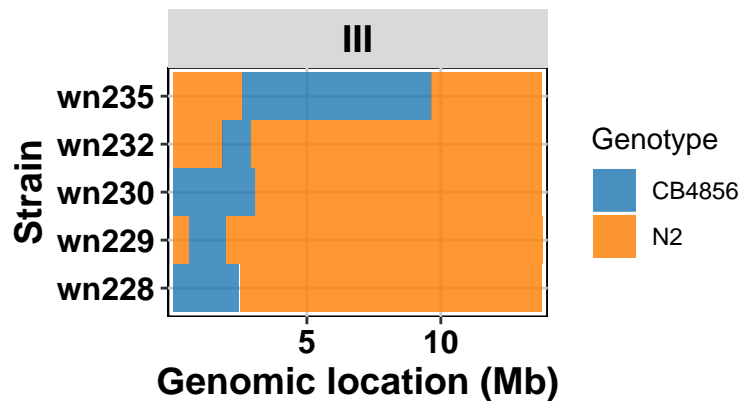**C**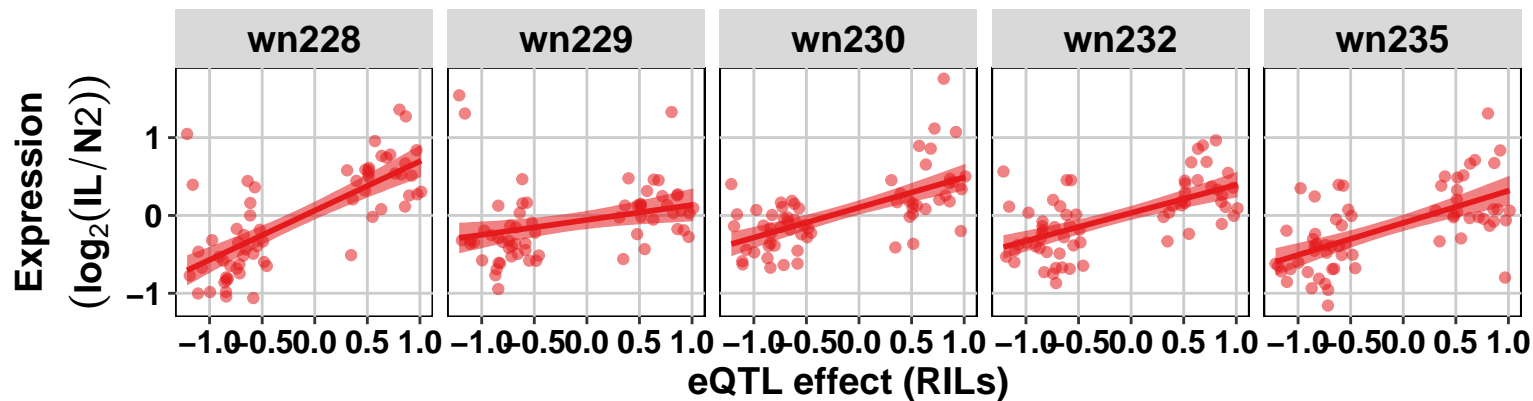

**A**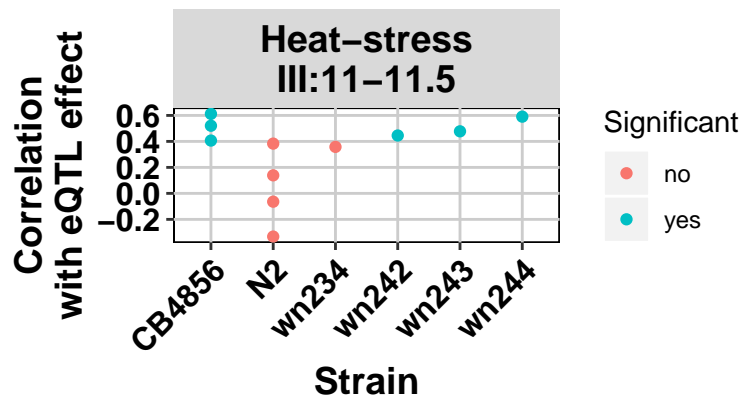**B**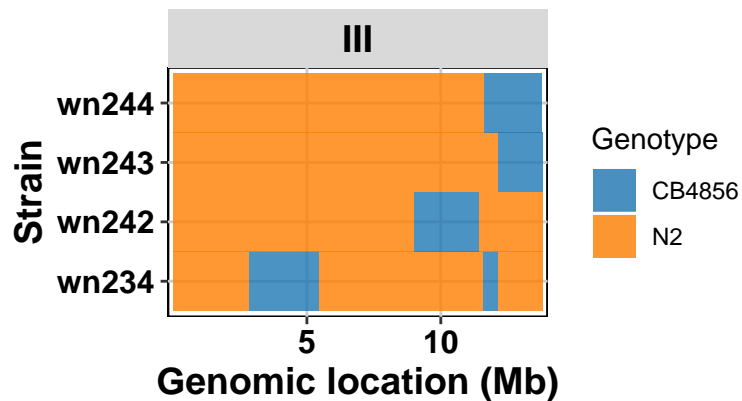**C**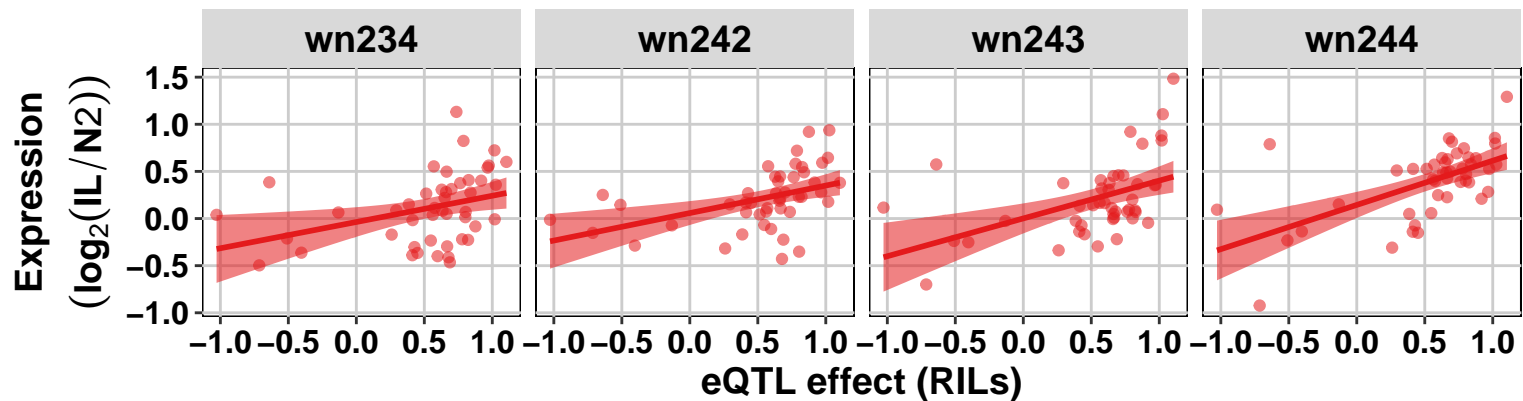

**A**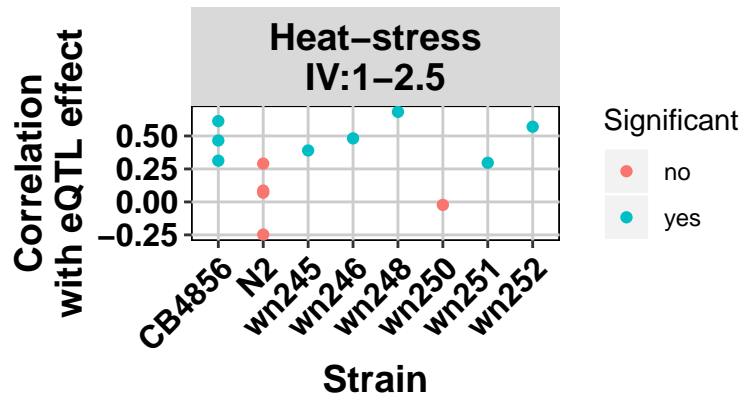**B**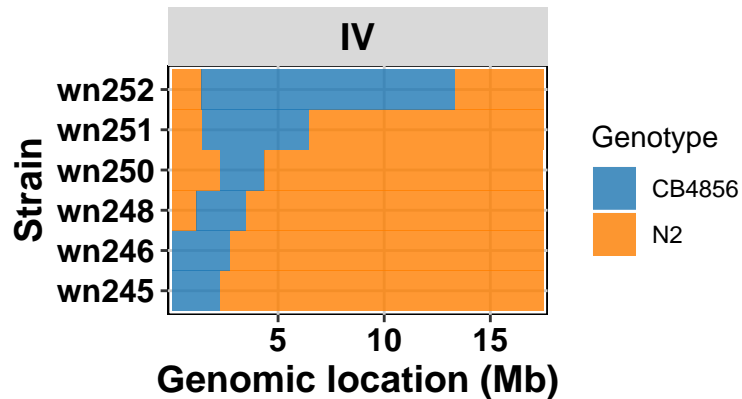**C**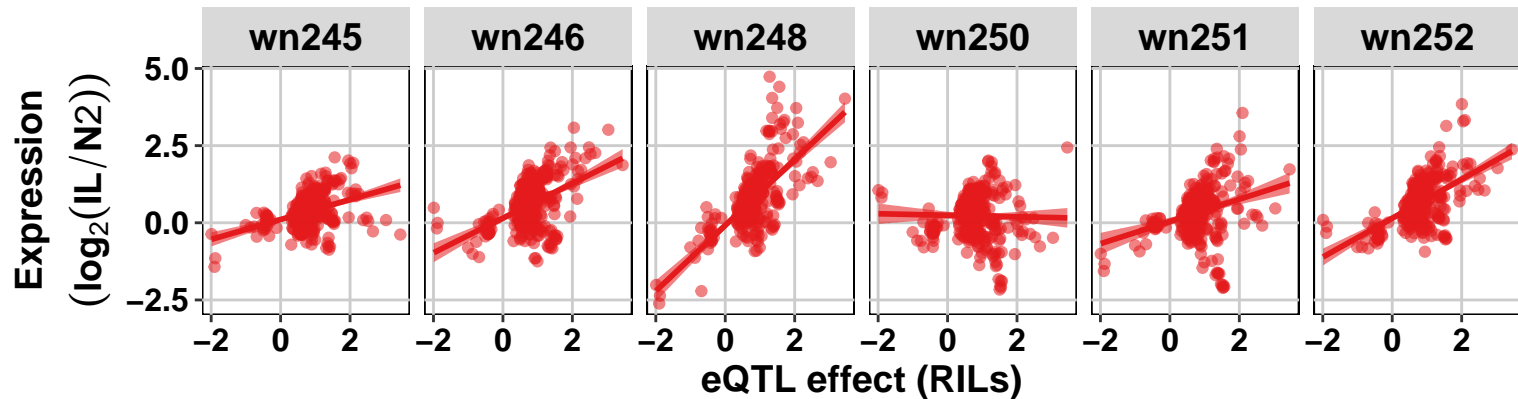

**A**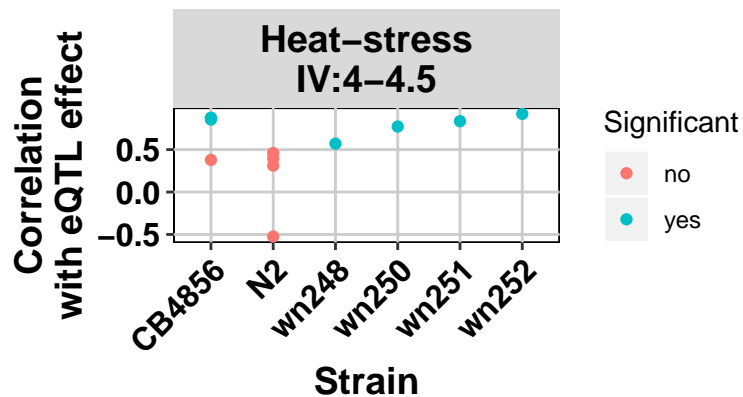**B**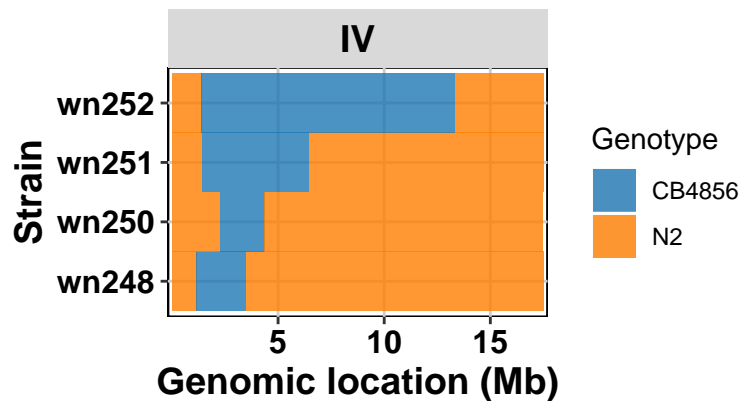**C**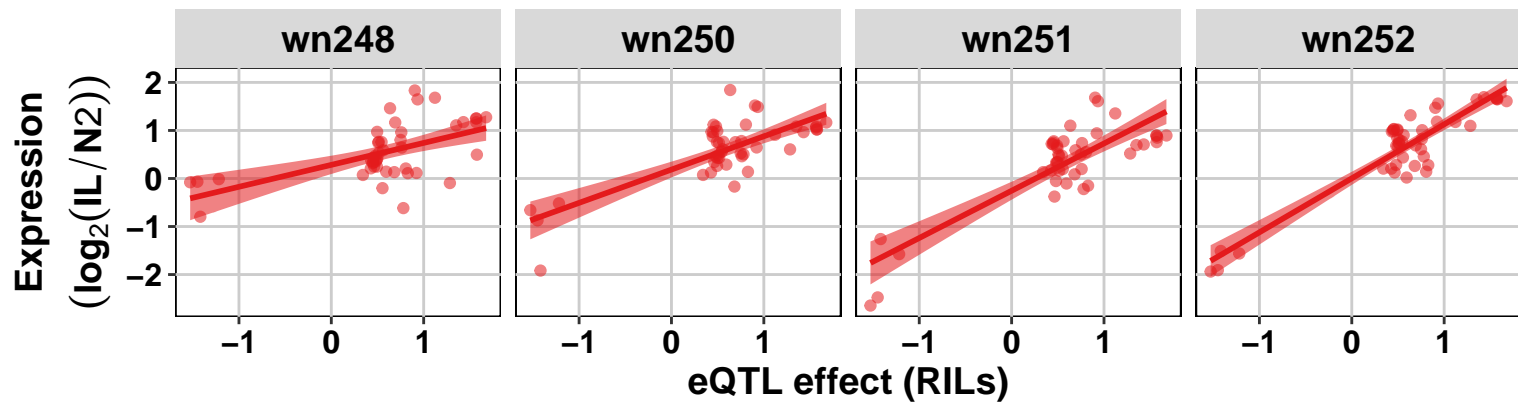

**A**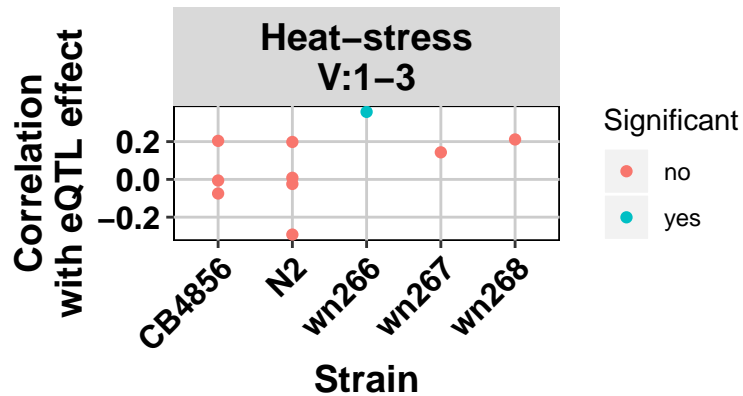**B**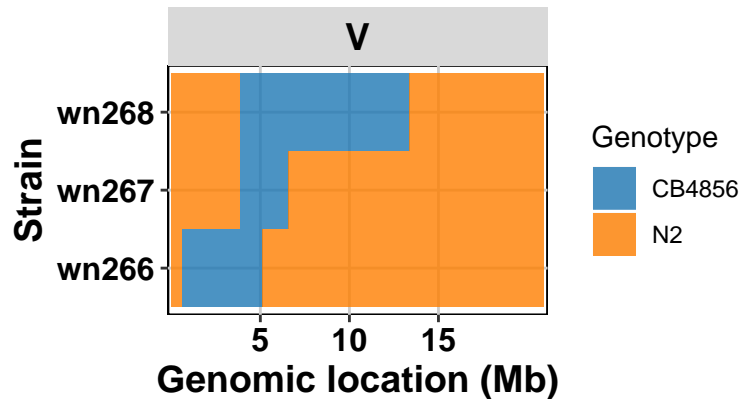**C**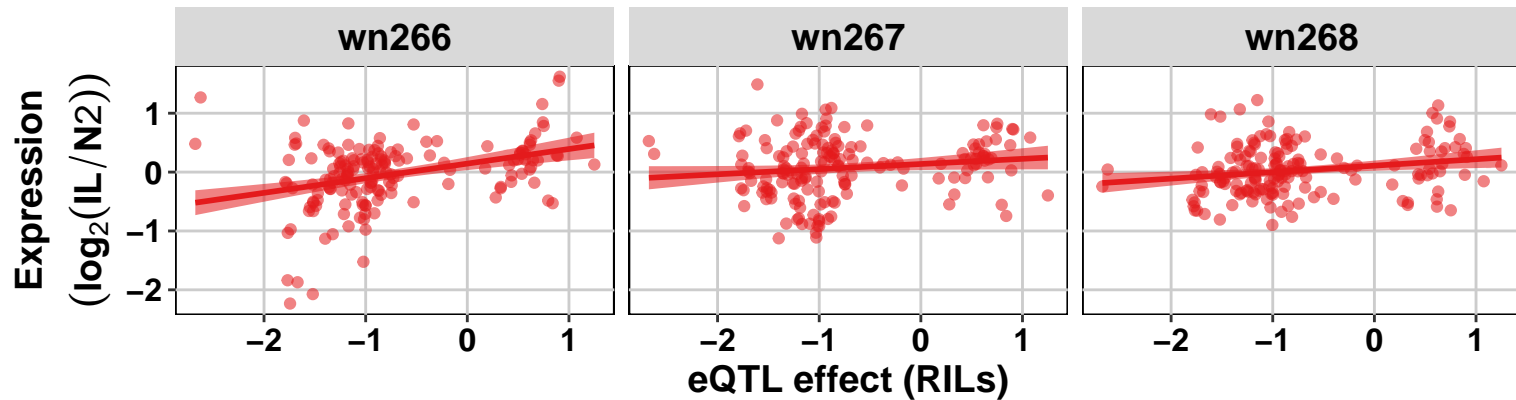

**A**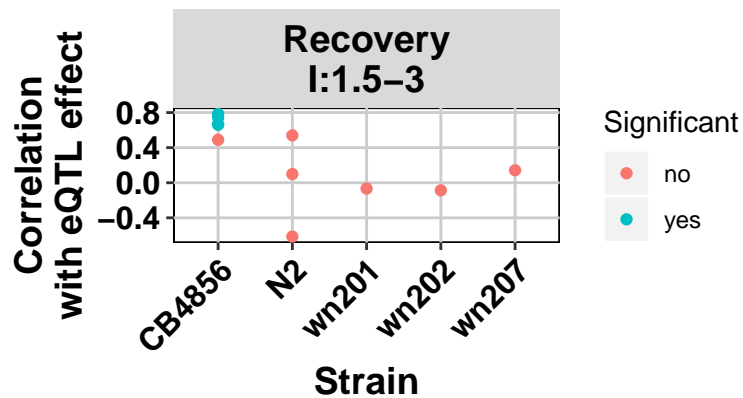**B**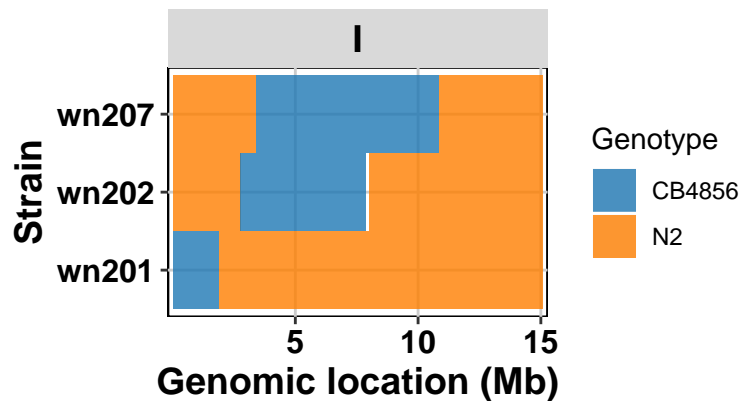**C**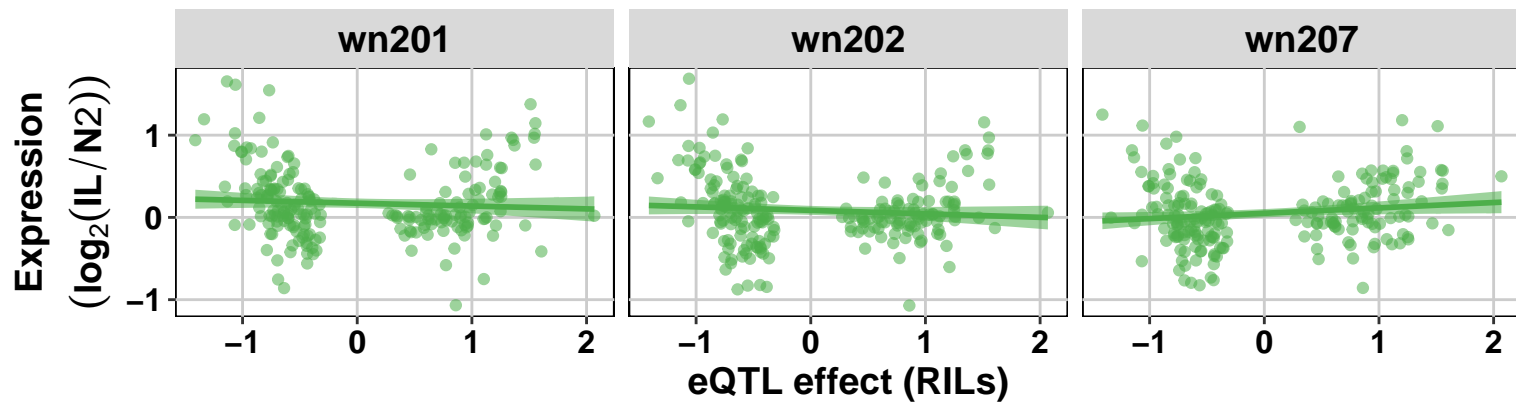

**A**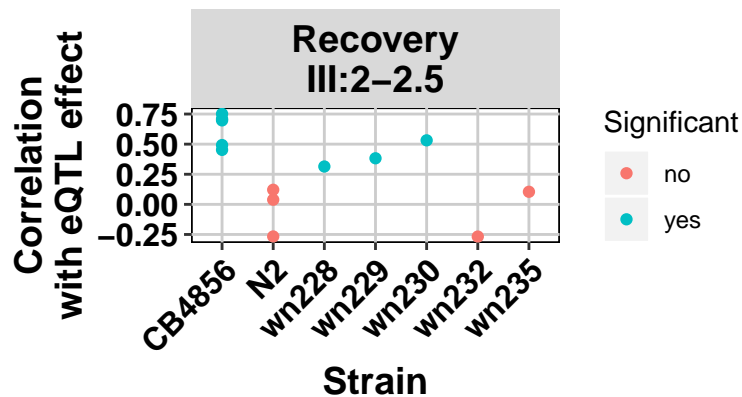**B**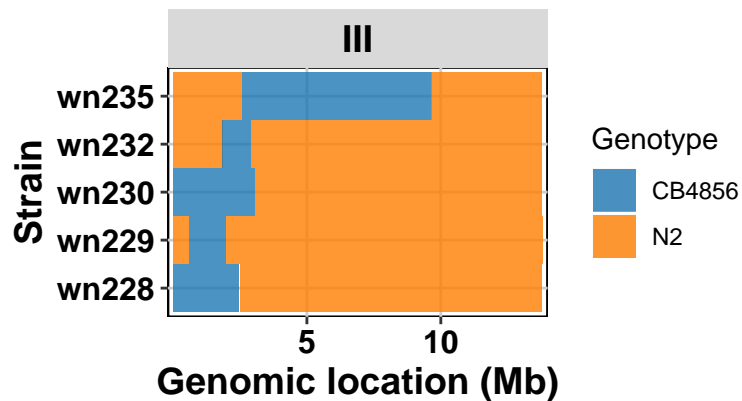**C**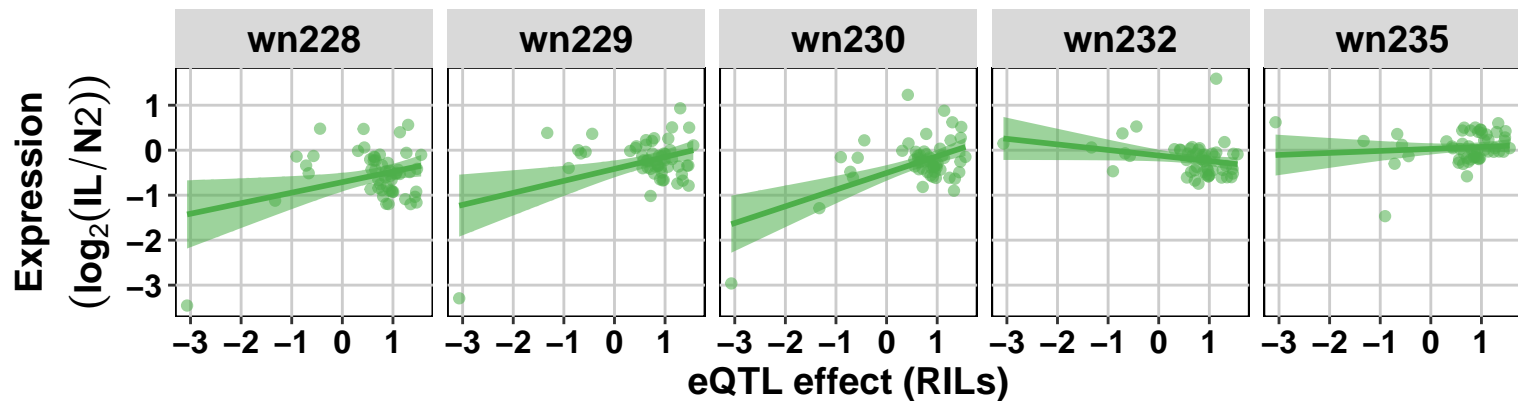

**A**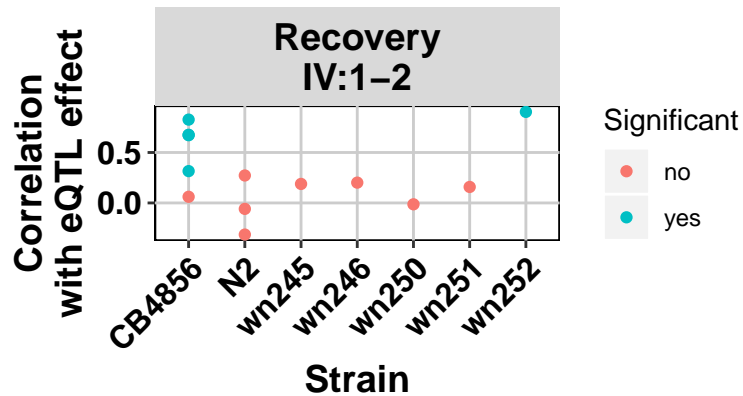**B**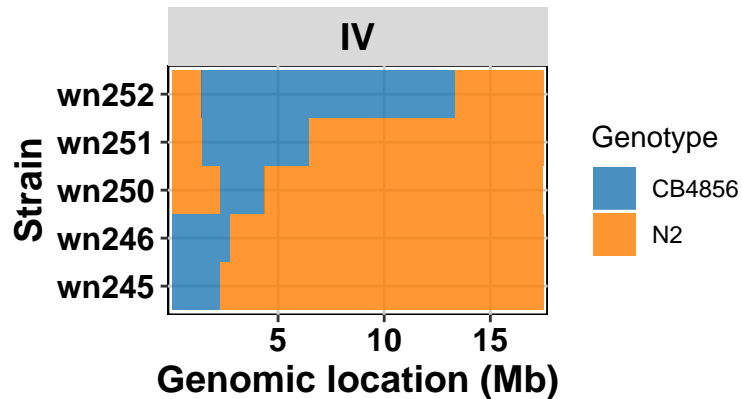**C**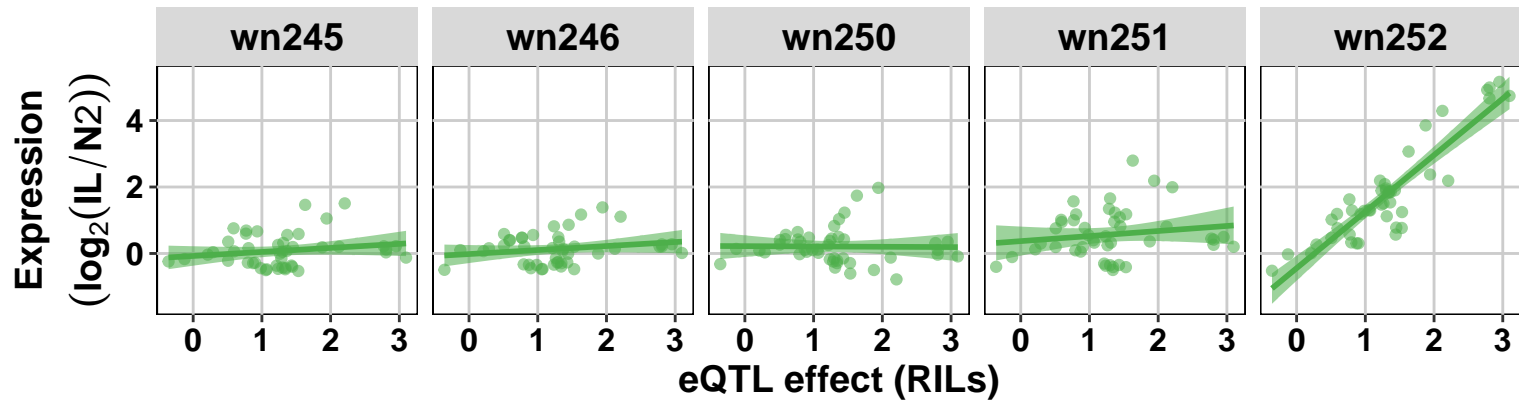

**A**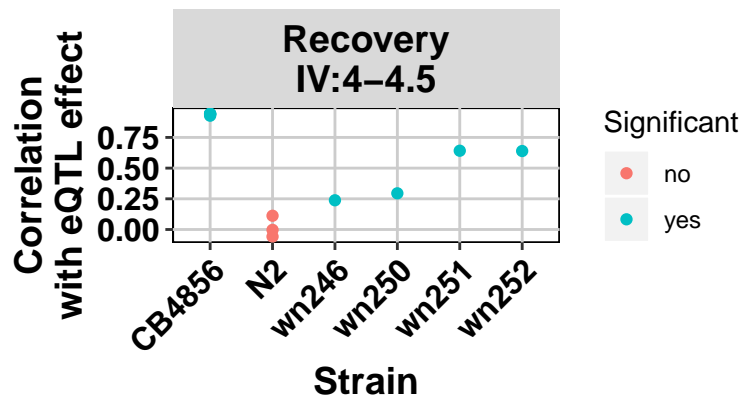**B**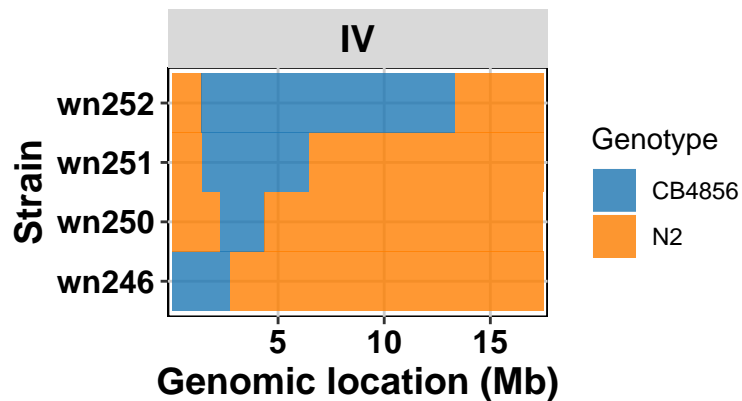**C**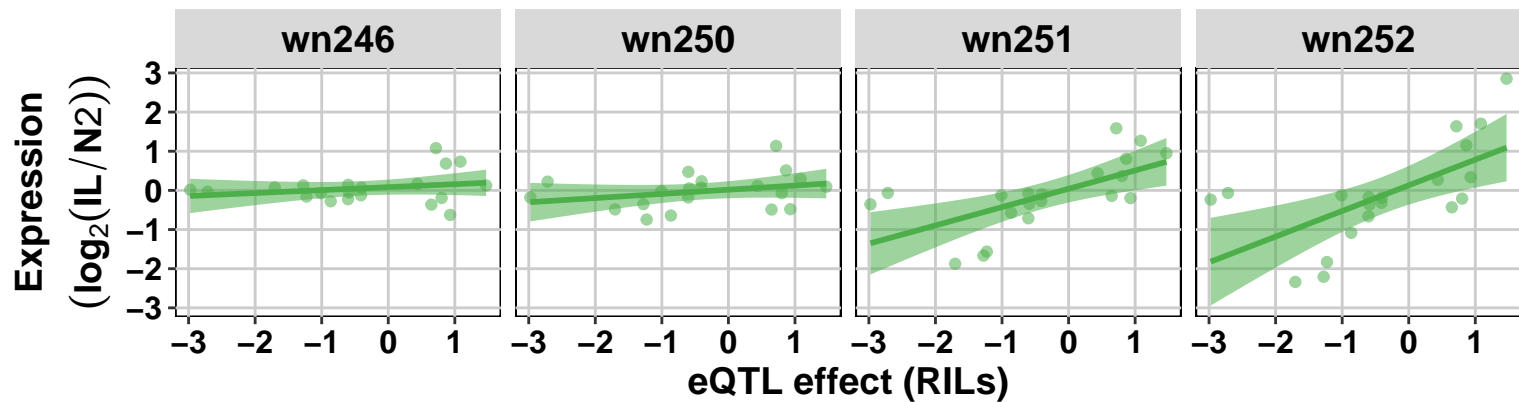

**A**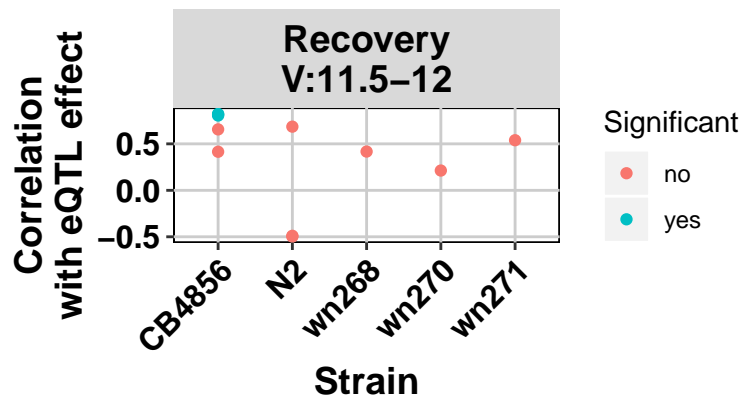**B**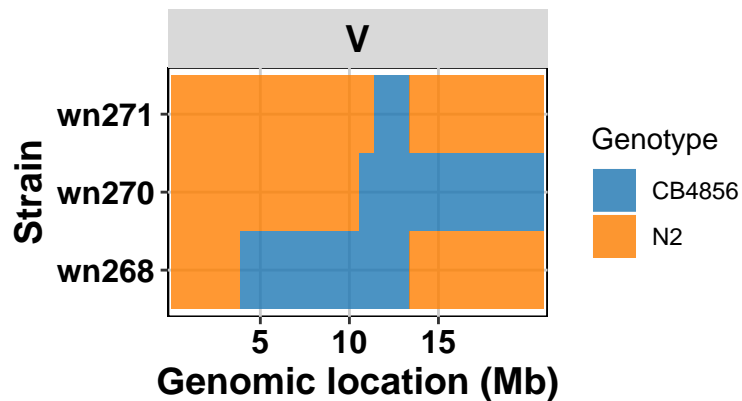**C**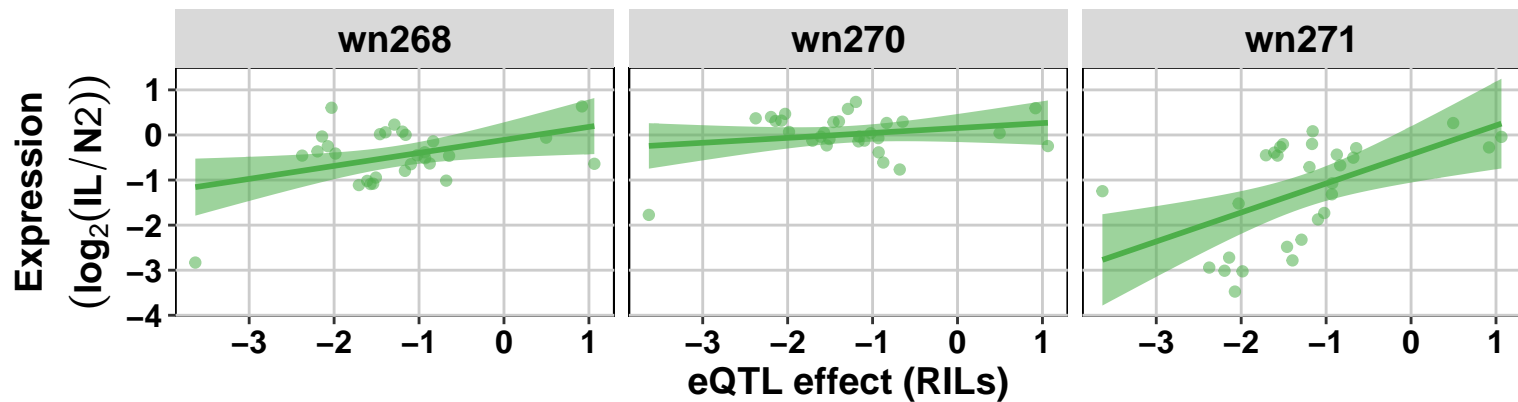

**A**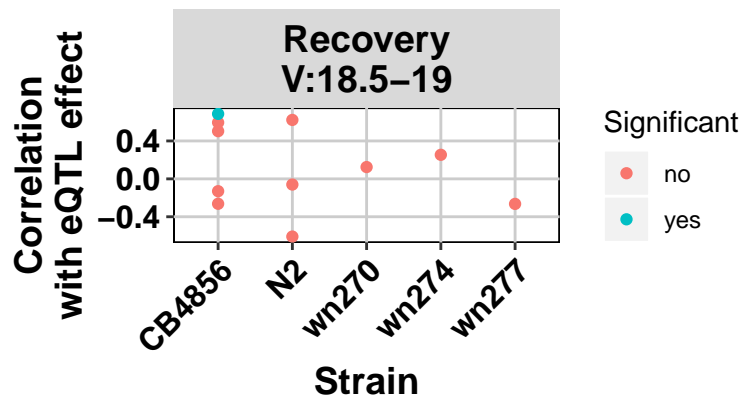**B**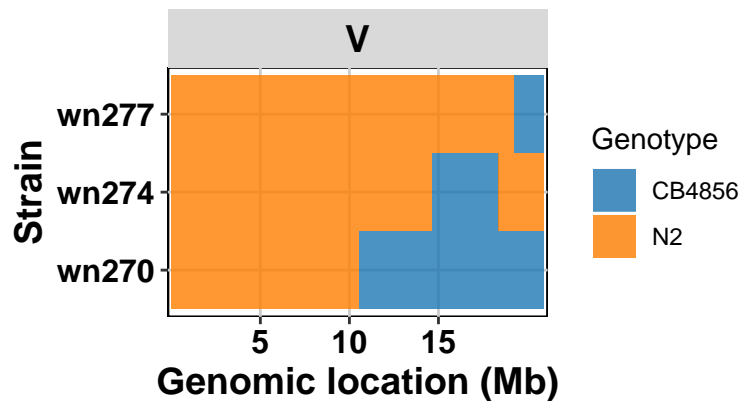**C**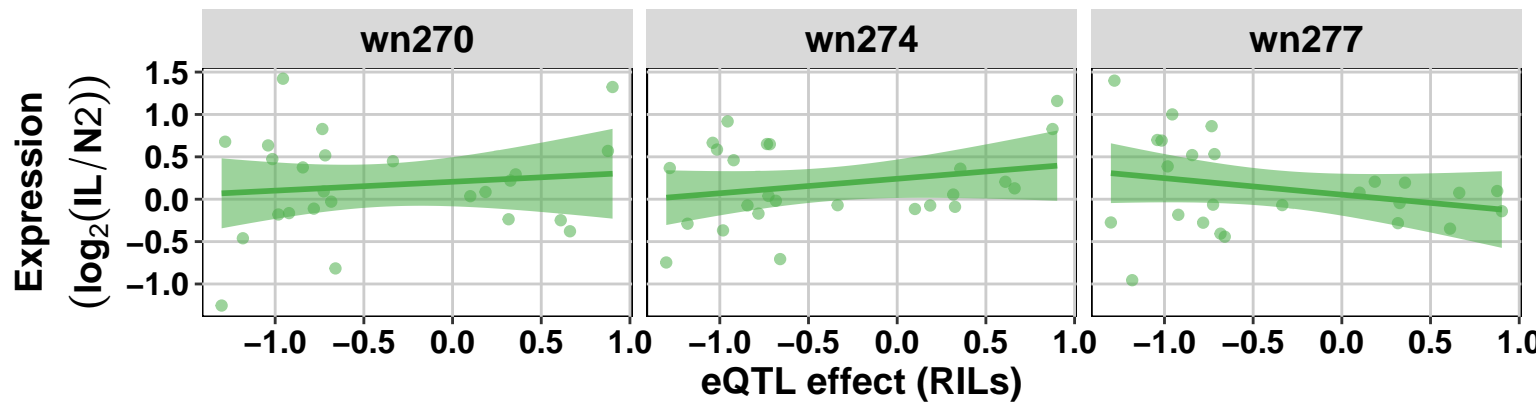

**A**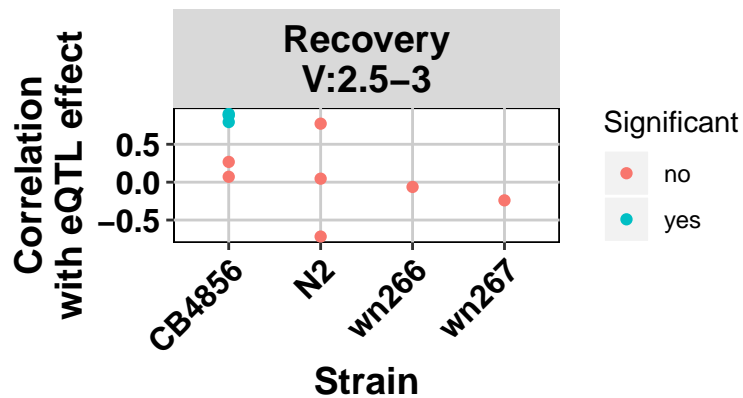**B**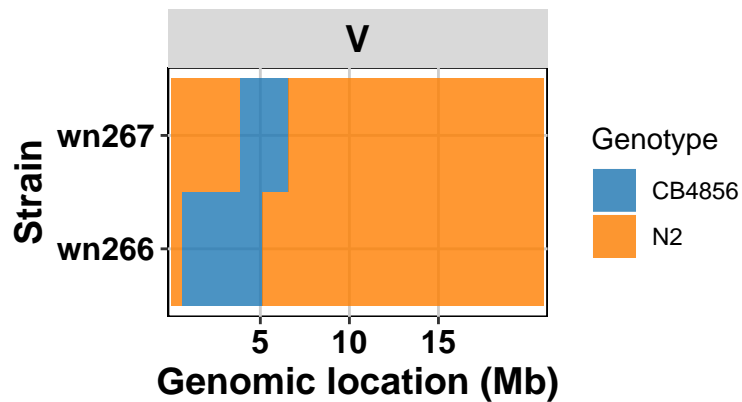**C**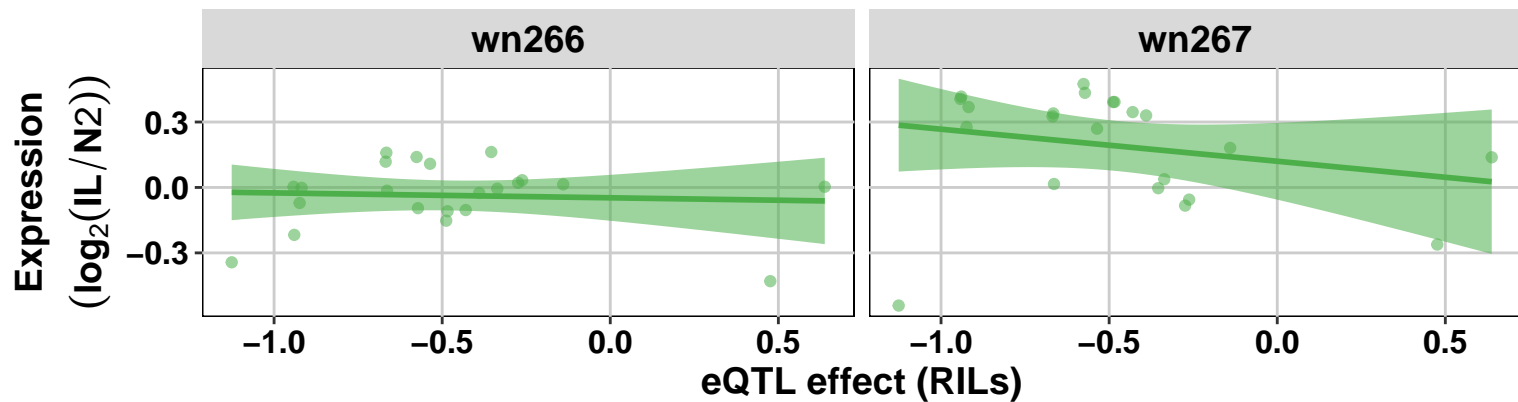

Supplement: Supplementary File 1 — Comparison of gene expression in ILs with eQTL in trans-bands identified in the RIL population. Per trans-band, a figure was constructed. In (A) the name of the trans-band is given at the top of the panel (e.g., chromosome I, 3.5–4 million bases) the correlation of the relative gene expression (RN2) was correlated with the eQTL effect for the ILs covering or nearby the trans-band. Significance was determined based on the highest correlation in the N2 strains, only strains with a stronger correlation than N2 were scored as significant. In (B) the genetic map of the ILs of the trans-band chromosome is shown. On the x-axis the genomic location (in million bases) is shown and on the y-axis the ILs. In (C) the actual correlation between expression in the ILs and eQTL effect in the RILs is shown. With on the x-axis the eQTL effect and on the y-axis RN2. Each dot represents a spot on the microarray with an eQTL in the trans-band investigated. The line indicates the estimated slope and the pale blue area around the line indicates the confidence interval of the fit. [file Data_Sheet_1.PDF]
